# Structures of the Apo and FAD-Bound Forms of 2-Hydroxybiphenyl 3-monooxygenase (HbpA) Locate Activity Hotspots Identified by Using Directed Evolution

**DOI:** 10.1002/cbic.201402701

**Published:** 2015-03-03

**Authors:** Chantel N Jensen, Tamara Mielke, Joseph E Farrugia, Annika Frank, Henry Man, Sam Hart, Johan P Turkenburg, Gideon Grogan

**Affiliations:** York Structural Biology Laboratory, Department of Chemistry, University of YorkHeslington, York, YO10 5DD (UK)

**Keywords:** biotransformations, FAD, flavoprotein, hydroxylation, monooxygenases

## Abstract

The FAD-dependent monooxygenase HbpA from *Pseudomonas azelaica* HBP1 catalyses the hydroxylation of 2-hydroxybiphenyl (2HBP) to 2,3-dihydroxybiphenyl (23DHBP). HbpA has been used extensively as a model for studying flavoprotein hydroxylases under process conditions, and has also been subjected to directed-evolution experiments that altered its catalytic properties. The structure of HbpA has been determined in its apo and FAD-complex forms to resolutions of 2.76 and 2.03 Å, respectively. Comparisons of the HbpA structure with those of homologues, in conjunction with a model of the reaction product in the active site, reveal His48 as the most likely acid/base residue to be involved in the hydroxylation mechanism. Mutation of His48 to Ala resulted in an inactive enzyme. The structures of HbpA also provide evidence that mutants achieved by directed evolution that altered activity are comparatively remote from the substrate-binding site.

## Introduction

**Scheme 1:**
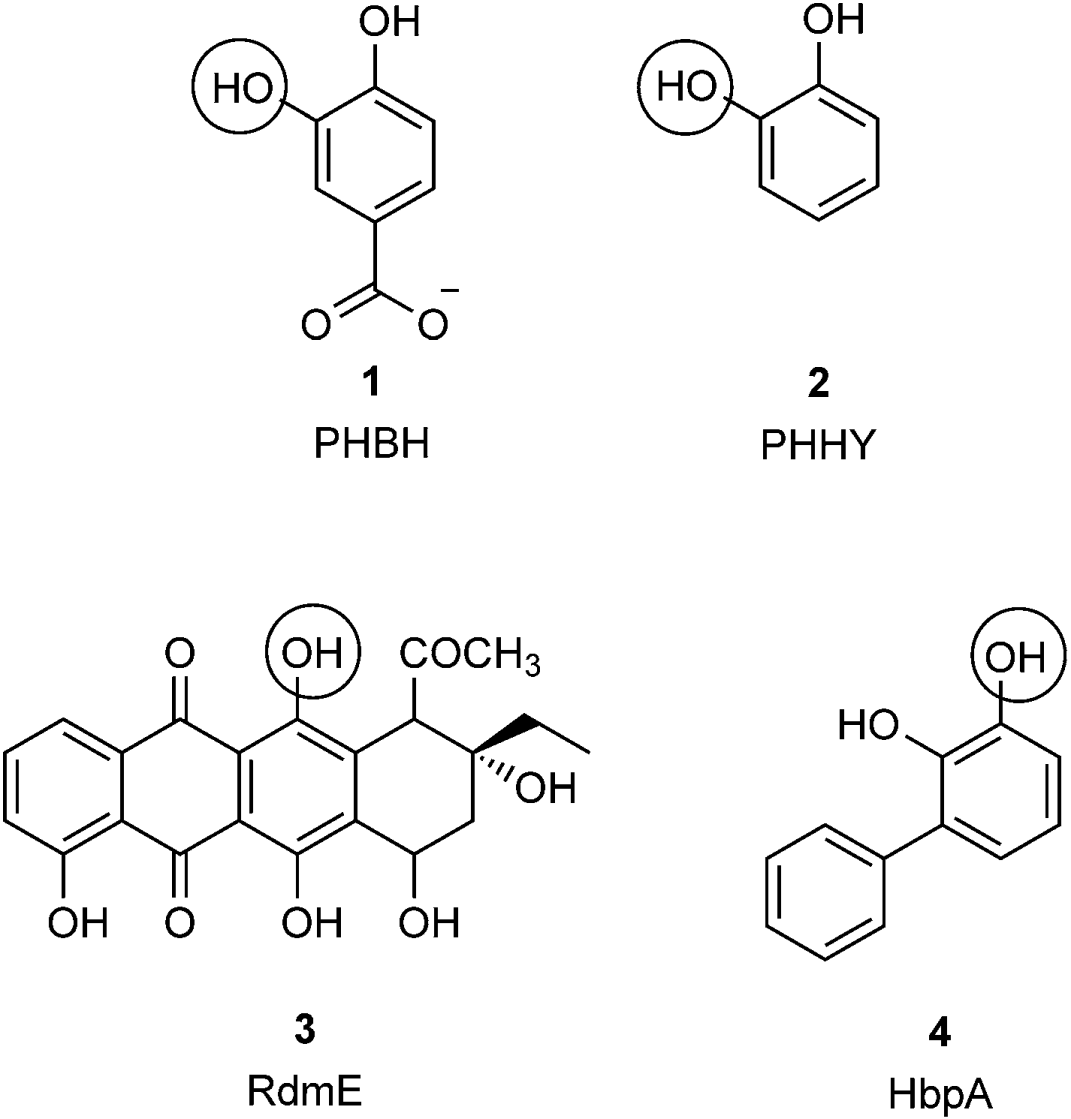
Products for some class “A” FPMO aromatic hydroxylases. The hydroxy group added by the enzyme indicated is circled. PHBH: *para*-hydroxybenzoate hydroxylase, PHHY: phenol hydroxylase, RdmE: aklavinone-11-hydroxylase, HbpA: 2-hydroxybiphenyl 3-monooxygenase.

Microbial flavoprotein monooxygenases (FPMOs) are involved in a host of important biochemical processes in a range of organisms, with many roles in catabolism and natural-product biosynthesis.[1,2] Owing to their ability to selectively functionalise organic molecules of interest, they also have great potential as biocatalysts for preparative industrial reactions.[3,4] A survey by van Berkel and Fraaije in 2006 divided the FPMOs into six sub-classes A–F.[3] Subclass “A”, which consists of the aromatic flavin-dependent hydroxylases, is an attractive group of enzymes from the perspective of preparative biocatalysis, as these enzymes catalyse the selective hydroxylation of aromatic substrates to phenols, the abiotic preparation of which requires harsh conditions and toxic reagents that are inconsistent with contemporary demands for sustainable chemical synthesis. The model enzyme from subclass “A” has been the NADPH-plus-FAD-dependent *para-*hydroxybenzoate hydroxylase (PHBH),[5] which catalyses the hydroxylation of the substrate to 3,4-dihydroxybenzoate (**1**, Scheme 1), and for which an X-ray structure[6] and mechanism[7] were first proposed in 1979.

PHBH has served as a model for structural and mechanistic studies in the aromatic hydroxylases ever since.[8] PHBH and other class “A” FPMOs are thought to catalyse aromatic hydroxylation reactions through a mechanism of two distinct phases. In the first, the nicotinamide cofactor NAD(P)H is used to reduce FAD. This reduction is stimulated by the binding of the aromatic substrate, which acts as an effector. In the second half of the reaction, reduced FADH_2_ reacts with molecular oxygen to produce a C4a-(hydro)peroxyflavin species that acts as the oxygenating agent in catalysis. Hydroxylation of the aromatic nucleus is thought to occur by electrophilic aromatic substitution, in which the hydroperoxide is the electrophile and the hydroxybenzoate aromatic ring is the nucleophile.[8] The nucleophilicity of the aromatic ring is increased by deprotonation of the phenolic hydroxy group by active-site residues. Extensive structural studies on PHBH[9–11] have also revealed that catalysis is characterised by a mobile flavin that exists in different conformations depending on the step in the catalytic cycle. For flavin reduction, the FAD is displaced away from the substrate binding site to the periphery of the enzyme (“out” position), where it can be reduced through interaction with NAD(P)H. In the presence of substrate, the flavin is located in the substrate binding site (“in” position). Reduced FADH_2_ in the “in” position is able to participate in both the reaction with oxygen, to form the hydroperoxide, and then the aromatic hydroxylation.

In addition to PHBH, other structurally and mechanistically related class “A” FPMOs that have been characterised range from phenol hydroxylase (PHHY), which forms catechol (**2**;[12,13] Scheme 1), to 3-hydroxybenzoate 6-hydroxylase[14] and biosynthetic enzymes including RebC, which is involved in the production of rebeccamycin,[15,16] and aklavinone-11-hydroxylase (RdmE), which hydroxylates the anthracycline precursor aklavinone (**3**).[17] In 1988, Kohler and co-workers reported the isolation of the bacterium *Pseudomonas azelaica* HBP1, which was able to employ as sole carbon source the fungicide 2-hydroxybiphenyl (2HBP).[18] The breakdown of the biaryl core was initiated by the *ortho-*hydroxylation of the substrate to yield 2,3-dihydroxybiphenyl (23DHBP). This aromatic hydroxylation was later attributed to a class “A” FPMO, 2-hydroxybiphenyl 3-monooxygenase (HbpA), which was isolated and characterised.[19] Mechanistic studies confirmed that HbpA shared many of the catalytic characteristics of class “A” FPMOs such as PHBH.[20] The gene encoding HbpA was cloned and overexpressed in the heterologous host *Escherichia coli*[21] to facilitate its application to the gram-scale hydroxylation of a range of 2-substituted phenols[22] and it has also served as a model for the activity of flavin-dependent hydroxylases under process conditions, including studies that investigated the stability of the purified enzyme in the presence of co-solvents such as methanol and decanol.[23] HbpA was also the subject of successful directed-evolution experiments that yielded mutants that were superior to the wild-type in their biotransformation of *tert-*butyl phenol[24] guaiacol and 2-*sec*-butylphenol,[25] displayed improved coupling of NADH oxidation to substrate hydroxylation, and also a mutant capable of the hydroxylation of indole.[26] In an effort to shed light on the structural consequences of evolution experiments, and also to provide further information on substrate recognition and mechanism in HbpA, preliminary X-ray crystallographic studies on the enzyme were performed.[27] Although crystals were obtained, a solution of the structure was frustrated by the short lifetime of the crystals within the X-ray beam; 20 crystals were required to provide a single dataset. In this report, using a new genetic construct and exploiting advances in X-ray data collection time, we have used single crystals of HbpA to determine its structure in two forms: an FAD-bound form that allows characterisation of the active site, and an apo form that, although lacking flavin, gives extra information on the location of residues and structure of mobile loops that are absent from the FAD complex. The structure of HbpA allows the beneficial mutation sites previously identified to be put into a tertiary structural context for the first time, and also provides a robust platform for further protein engineering of this useful enzyme.

## Results and Discussion

### Quality of the models

Sub-cloning of the *hbpA* gene and its expression from the YSBLIC-3C vector resulted in a protein that is 21 residues longer than the native protein by virtue of a spacer that leads from the N-terminal methionine to the histidine tag, and also incorporates a C3 protease cleavage site. The tag was not removed prior to crystallisation. Protein resulting from this construct gave crystals of both a different space group (*P*1 rather than *C*2) and cell dimensions from those obtained by Meyer and co- .[27] These crystals had sufficient longevity within the X-ray beam for a complete dataset to be collected by using one crystal in each case for the apo form of the enzyme and the FAD complex. The structure of the apo form was obtained first, after which it was determined that soaking with 1 mm FAD was necessary to obtain structural data in which FAD was at full occupancy within each active site of HbpA. In the apo form, the amino acid backbone was complete from residues Ser4 to Arg228, Ala239 to Trp254 and Glu268 to Arg565. The FAD complex was less complete in each subunit, with density of a quality sufficient for building present from Ser4 to Gly194, Ser203 to Asp211, Asp222 to Arg228, then Gly237 to Met243; Trp250 to Trp254 then Glu268 to Arg585. In each case, the breaks in electron density corresponded to loop regions between the seven β-strands of the substrate binding domain **D2**, as described in detail below.

### Structure of the HbpA monomer

**Figure 1:**
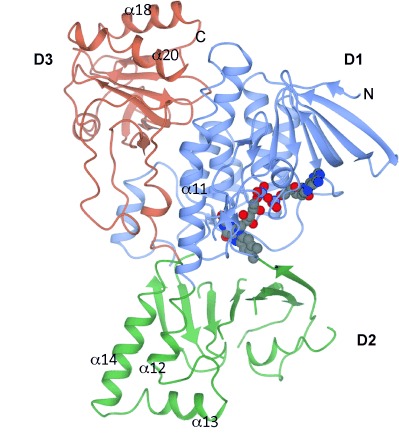
Monomer of HbpA FAD complex shown in ribbon format. Selected α-helices are labelled. FAD is shown in sphere format (carbon atoms in grey), with the isoalloxazine ring bound at the interface between domains 1 and 2. N and C indicate the amino- and carboxy terminus of the monomer.

The monomer of HbpA (Figure 1) broadly resembles the class “A” FPMO aromatic hydroxylases PHHY, RebC and RdmE of established structure and consists of three domains: an FAD nucleotide binding domain (**D1**) containing residues 4–76, 96–197 and 295–374; a “middle” domain[17] (**D2**), composed of residues 77–95, 375–427 and 198–294; and a C-terminal domain (**D3**) incorporating a thioredoxin-like fold formed from residues 428–585.

The closest structural homologues of HbpA, as revealed by analysis using the DALI server[31] are the flavin-dependent monooxygenase RebC, from *Lechevalia aerocologines* (PDB ID: 4EIP; 28 % sequence identity; *Z*-score 39.8; rmsd 2.6 Å over 489 backbone Cα atoms),[15,16] the aklavinone-11-hydroxylase (RdmE) from *Streptomyces purpurascens*[17] (PDB ID: 3IHG; 35 % sequence identity; *Z*-score 39.2; rmsd 2.2 Å over 474 backbone Cα atoms) and the phenol monooxygenase (PHHY) from *Trichosporon cutaneum* (*Z*-score 35.2; 1PN0/1FOH; 22 % sequence identity; rmsd 2.9 over 476 backbone Cα atoms).[12,13]

The FAD binding domain, **D1**, is a Rossman-type domain with a central mixed β-sheet of six strands, bordered by an additional sheet of four strands, on the side nearest the enzyme periphery, and four substantial α-helices (α1, α6, α10 and α11) at the interface with the thioredoxin domain, **D3**. The loop of amino acids 35–52 between strand β2 and helix α2 runs behind, and provides close contacts with, the isoalloxazine ring of FAD. HbpA lacks the large additional loop region in **D1** that is present in PHHY (formed by residues 170–210), which is also absent in RdmE and which is thought to constitute a “lid” over the active site of PHHY.[12] The middle domain, **D2**, consists of a large seven-stranded mixed β-sheet that provides the floor of the substrate binding site. Poor density in the loops connecting these strands accounts for the most of the chain breaks in both the apo and especially the FAD-complex structures listed above and is indicative of a higher level of mobility in this region. The sheet is adjacent to a helix-turn-helix-turn-helix subdomain formed by residues 325–427 of domain **D2**, which incorporates helices α12, α13 and α14 (Figure 1) and which constitutes one of the major structural differences between HbpA, RebC, RdmE and PHHY. In PHHY, this subdomain is smaller, lacking the equivalent of α13, and in RdmE is almost absent, replaced by a loop of only 15 amino acids (RdmE 373–388). This large sub-domain in HbpA is almost certainly important for dimer stabilisation within the HbpA tetramer (vide infra). Domain **D2** is connected by a long loop from amino acids 428-471 to the thioredoxin domain **D3**, which, although of unknown function in FPMOs, is involved in many reciprocal contacts with the helix-turn-helix-turn-helix region 325–427 in domain **D2** of the neighbouring monomer.

### Structure of the HbpA tetramer

**Figure 2:**
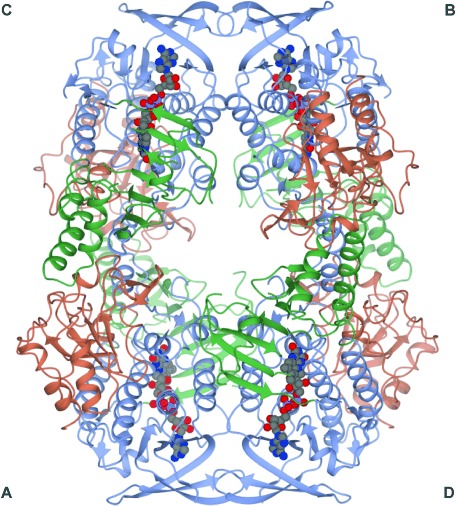
Structure of the HbpA tetramer ABCD, shown in ribbon format with subunits labelled and domains D1, D2 and D3 of each subunit coloured in light blue, green and coral, respectively, as in Figure 1. FAD molecules are shown in sphere format, with carbon atoms in grey.

Data on the tetrameric assembly observed in the crystal structure (Figure 2), which were provided by PISA analysis,[32] appear to provide good evidence for a physiological tetramer, as suggested previously:[19] the contact area between monomers **A** and **C** is 1243 Å^2^, with a Δ*G* value for this interaction of −8.5 kcal mol^−1^; the values for the dimer formed by monomers **A** and **D** were 1037 Å^2^ and −12.7 kcal mol^−1^. There are ten hydrogen bonds and 13 salt bridges between the **AC** pair, and between the **AD** pair are 11 and eight, respectively. The **AC** dimer is held together by reciprocal interactions between domain **D3** and helix α14 of domain **D2**, and also by hydrogen bonds between the Gln102 side chains in the short helices α5 that connect domains 1 and 2. The **AD** pair is held together by extensive reciprocal interactions between the N-terminal four-stranded β-sheets and helices α3 and α6 of domain **D1**. There is a large cavity at the centre of the tetramer into which the side chains of several hydrophilic amino acids of each monomer, including Glu75 and Tyr76, are projected.

The aromatic hydroxylases PHHY[12] and RdmE[17] are reported to exist in solution as a homodimer and a monomer, respectively. The determinants of the **AC** homodimer interactions in PHHY (1FOH) are very different from those of the **AC** dimer found in HbpA; in the PHHY dimer, much more extensive reciprocal interactions exist between domains 2 and 3, giving a contact area of 1900 Å^2^, as a result of a relative closure of the AC subunits relative to HbpA. Dimer formation appears to be assisted in PHHY by a large movement of the loop 170–210 (absent in HbpA) in domain 1 in only one of the monomers that brings this loop into close contact with its dimer partner. Interactions between the more stable dimer pair **AD** of HbpA (as indicated by Δ*G* values) are actually governed by reciprocal interactions between the **D1** domains, which make comparatively few interactions in the PHHY AC dimer, as a result of the extended loop. The structure of **D1** appears to be quite well conserved between HbpA and RdmE, so the reason for the failure of the latter to oligomerise in an equivalent way to the HbpA **AD** dimer is not clear, but the inability to form an HbpA **AC**-type dimer might be attributed to its lack of a substantial subdomain within **D2**.

### Comparison of the apo and FAD structures

Although lacking the FAD coenzyme, the apo structure of HbpA has proved useful in providing information on secondary structural elements and residues that are missing from the FAD-complex structure. The overall rmsd between the two structures is low, at 0.85 Å over 538 backbone Cα atoms, as would be expected, but there is a pronounced shift in the relative orientation of domain **D2** away from domain **D1** as a result of FAD binding; this results in a more open active site. In the apo structure, the backbone Cα atoms of Arg242 and Met243 on strand β15 in **D2** closest to FAD, for example, each move 2.5 Å away from the binding site in a superimposition of the two structures. The relative shift in domains is coincident with an absence of some electron density in regions in the FAD complex compared to the apo structure. These include density for the loop 195–203 that connects domains **D1** and **D2** as well as additional loops between the seven β-strands of domain **D2**, including region 245–249 between β15 and β16 and region 213–219 between β13 and β14, which features the short helix α8 in the apo structure. The reasons for the mobility of these loops on FAD binding are unclear; they correspond neither to substrate entry channels—as proposed, for example, for RdmE[17]—nor to the region of the protein that would move as a result of FAD moving to the “out” position, as also observed in RdmE. The structural reorganisation upon FAD binding is also manifested in the orientation of the loop between residues 36 and 49 between β2 and β3, which moves to accommodate the isoalloxazine ring of FAD.

### FAD and NADH binding

**Figure 3:**
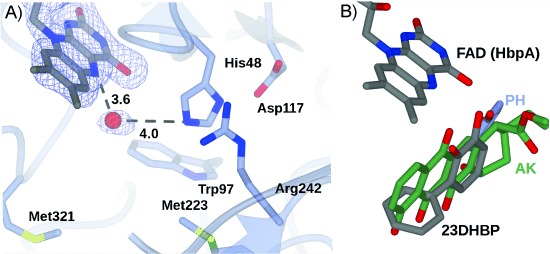
A) Detail of HbpA structure in the FAD binding domain, illustrating the water molecule (red sphere) closest to the C4a atom of the FAD and acid/base residues including His48, which forms a dyad with Asp117, and Arg242. The side chains of Met223 and Met321 contribute to the hydrophobic environment within the active site. Electron density corresponds to the omit (*F*_o_−*F*_c_) map contoured at a level of 3 *σ* and refined in the absence of FAD, the atoms of which have been added subsequently for clarity. B) Superimposition of the structures of RdmE and PHHY (1FOH) with all atoms bar those of the relevant ligands, AK and PH, respectively, removed. The nucleophilic carbon atoms, which wil be hydroxylated in each case, superimpose with each other. These structures have been used to inform the modelling of the HbpA active site with the product 23DHBP (grey).

In the FAD complex, the flavin is bound within HbpA in the “in” position described for other class “A” FPMOs. As a consequence of the relative movement of domains **D1** and **D2**, residue Trp293 on strand β17 has moved to shield the isoalloxazine ring of FAD from the solvent. The ADP moiety of FAD is bound in a pocket near the surface of **D1**. The adenine ring is stacked against the side chain of Arg37, with the exocyclic NH_2_ bonded to the backbone carbonyl of Tyr144. The adenine ring also makes hydrogen bonds with the peptidic NH groups of Arg37 and Tyr144. The ribose hydroxy group is H-bonded to the side chain of Asn36, and the diphosphate bonds to the peptidic NH groups of Ala17 and Asp313, and also to water molecules. The ribitol side chain interacts with the side chains of Asp313, Arg46 and Gln120. The tricyclic isoalloxazine ring of FAD is bound deeper within the protein at the base of domain **D1** and near the interface with **D2**, with the isoalloxazine ring stacked between loop 46–49 preceding strand β3, and loop 319–324 between β21 and α10. The loop above the flavin provides the side chains of Ser47, which is directly above the central FAD ring, and His48, which is discussed in more detail below. The loop beneath the flavin provides Pro320 and Met321 (Figure 3 A), which also help to form one wall of the substrate binding pocket, a large hydrophobic cavity whose other side formed by the seven-stranded β-sheet of domain **D2**.

The sheet of domain **D2** provides many hydrophobic side chains to the active site pocket, including Trp97, Met223 and Trp225. Among the hydrophobic side chains of the active site, there are also two hydrophilic residues, Arg242 and His48, which are situated to the front of the plane of the isoalloxazine ring (Figure 3 A). The imidazole ring of His48 is 5.5 Å from the C4a atom of FAD at which the hydroperoxide is formed. It is also 4.0 Å from a water molecule to the front and beneath the FAD ring that is well placed to mimic the hydroxy group of the hydroperoxide. Putative roles for His48 in the mechanism of HbpA are discussed in the context of a model complex structure below.

The structure of HbpA also allows a comparative analysis of the cofactor binding loop, which contains residues that assist in discriminating between the nicotinamide cofactors NADH and NADPH. In NADPH-dependent PHBH, specificity for the phosphorylated cofactor is thought to be determined largely by Arg33, Tyr38 and Arg42, with replacement of Tyr38 by an acidic residue leading to a shift in activity toward NADH.[33] The equivalent residues in HbpA: Arg37, Ser40 and Ser42 clearly provide a less positively charged environment for the NADPH phosphate, but there are no close structurally homologous acidic residues that suggest that NADH specificity in HbpA is achieved through carboxylate–ribose hydroxy interactions.

### Comparison of the HbpA active site with FPMO homologues PHHY and aklavinone-11-hydroxylase RdmE

Determination of the structure of HbpA allows its active site to be compared with that of its closest structural and functional homologues in an effort to distinguish the determinants of mechanism and substrate specificity in the enzyme. Despite numerous attempts at both co-crystallisation and soaking with both 2HBP and 23DHBP, no ternary complex of HbpA was forthcoming. We therefore sought to model the product into the active site of the enzyme by using PHHY and RdmE as the bases. Interestingly, in substrate complexes for both PHHY and RdmE, the nucleophilic carbons of the substrate to be hydroxylated superimpose very well (Figure 3 B), at a distance of approximately 5.3 Å and an angle of approximately 60° down from the plane of the FAD isoalloxazine ring. This, in addition to the orientation of the polycyclic ring system in RdmE, has provided anchoring positions for modelling the HbpA product 23DHBP into the substrate-binding pocket of HbpA.

It might be expected that some conservation of active-site residues between PHHY and HbpA would be observed, given the formal similarity of their substrates; of the hydrophobic side chains in the active site, for example, Met80 (PHHY) is conserved as Met77 (HbpA). However, for protic residues that interact directly with the phenol hydroxy group in PHHY, and have thus been implicated in mechanism,[12] Asp54, which is thought to assist in phenolate stabilisation, is replaced in HbpA by His48, and Tyr289, which is thought to act as the proton donor to the FAD peroxo-anion is replaced by Ala240. Given these differences, it is clear that, despite similarities in substrate structure, different mechanisms of substrate orientation and intermediate stabilisation operate in HbpA.

A comparison of the active site of HbpA with that of RdmE,[17] is also informative in that, in common with HbpA, this enzyme catalyses the hydroxylation of a polycyclic aromatic substrate. The recognition of a larger hydrophobic substrate is reflected in the hydrophobic make-up of the substrate binding channel in both enzymes. In the HbpA model product complex, the phenyl ring of the product is accommodated in a hydrophobic pocket formed by the side chains of Pro320, Met321, Trp225 and Met223, similar to the binding mode of the polycyclic ring system of aklavinone in RdmE, although the phenyl ring is rotated 90° relative to the phenol (Figure 3 B). The phenol is in a hydrophobic pocket formed by Ile49 and Trp97 near the pyrimidinedione ring of FAD.

As with PHHY, the aklavinone substrate is also thought to be activated by deprotonation in RdmE, which, in this case, is accomplished by Tyr224, which forms a hydrogen bond with the C6 hydroxy group of aklavinone *para* to the C11 atom. If deprotonation of the C2 hydroxy group of the substrate is also to be a significant step in the HbpA mechanism, the only candidate residue for deprotonation is His48. However, in the product model complex of HbpA, His48 is hydrogen bonded to the new C3-hydroxy group of the product. In the HbpA–FAD complex, the His48 NE2 atom is 4.0 Å from a water molecule that is itself 3.6 Å from the FAD C4a atom, and ideally placed to mimic the hydroxy group of a hydroperoxide intermediate. It might be therefore that His48 is the proton donor to the nascent hydroperoxide, the role thought to be fulfilled in PHHY by Tyr289. Interestingly, His48 forms a clear dyad with Asp117 in the second shell of residues around the active site. The only other hydrophilic residue that is close to the FAD is Arg242; the terminal guanidinium group is 8.2 Å from the FAD C4a, but also only 3.6 Å from the imidazole ring of the His48 side chain.

### The His48Ala mutant of HbpA is inactive for the hydroxylation of 2HBP

The location of His48, Asp 117 and Arg242 within the active site prompted us to make site-directed mutants of HbpA featuring alanine at these positions in order to test the effect of these mutations on the ability of the enzyme to bind and to hydroxylate 2HBP. Each of the mutant genes encoding His48Ala, Asp117Ala and Arg242Ala was expressed at similar levels in the soluble fraction as compared to the wild-type (WT). The apparent *K*_m_ values (*K*_m app_) for 2HBP for each of the mutants were then determined (Table 1) by UV spectrophotometry, measuring the oxidation of NADH at 340 nm at increasing substrate concentration, as described by Meyer et al.[25] Each mutant displayed Michaelis–Menten-type kinetics with 2HBP, with the WT *K*_m app_ value of 3.3 μm being of a similar order to that determined by Meyer et al. (2.6 μm).[25] The *K*_m app_ of His48Ala was approximately the same as for the WT, but Asp117Ala and Arg242Ala, displayed values approximately 1.7 times greater than WT in each case. Each mutant was clearly able to oxidise NADH. The maximum rate of NADH oxidation in these experiments, recorded in the presence of saturating concentrations of 2HBP (25 μm) was 0.02 s^−1^ for the WT enzyme, but was reduced approximately fourfold for the His48Ala and Asp117Ala mutants, and eightfold for the Arg242Ala mutant.

**Table 1:** Apparent *K*_m_ values for the 2HBP and NADH oxidase activity of HbpA variants.

	HbpA variant			
	WT	His48Ala	Asp117Ala	Arg242Ala
*K*_m app_ (HBP) [μm]	3.3±0.2	3.1±0.4	5.0±0.8	4.8±1.6

However, the rate of NADH oxidation as approximately the same for all variants in the absence of substrate; perhaps this indicates that the ability of 2HBP to act as an effector is compromised in each of these mutants.

The rates of NADH oxidation are often not indicative of the catalytic performance of HbpA with respect to substrate hydroxylation, however, owing to the inefficiency of electron-transfer steps between cofactors and substrate in the mechanism (“decoupling”).[25] The hydroxylation abilities of WT HbpA and mutant enzymes were therefore assayed by using the appearance of product on HPLC, as described in the Experimental Section. When WT HbpA was incubated with 0.5 mm 2HBP and NADH in threefold excess, the oxygenation to 23DHBP was complete after 2 h. No conversion to product was obtained for reactions containing either the His48Ala or Asp117Ala mutants. For the Arg242Ala mutants, approximately 15 % conversion to the product was obtained after 2 h. The results appear to suggest that both His48 and Asp117 are essential for the oxygenation reaction to occur, but that Arg242 is not. The precise role of His48 as a possible proton donor awaits the outcome of other experiments, including the determination of an HbpA structure in the presence of the substrate or product. The role of Asp117 might be either merely to orient the correct tautomer of the His48 imidazole ring for catalysis, as in ribonuclease A,[34] or to reduce the *p*K_a_ of the histidine NE-2 proton, thereby assisting its function as a catalytic acid. The role of Arg242 is unclear, but the higher *K*_m app_ observed for this mutant, coupled with the mobility of the domain bearing this residue (as revealed by comparison with the apo structure), suggests that interaction between this residue and the substrate cannot be ruled out. Although the precise role of His48 in catalysis remains unconfirmed, its identification as the major protic residue near the C4a atom now permits more thorough investigation of its mechanistic role.

### Location of activity hotspots in HbpA

**Figure 4:**
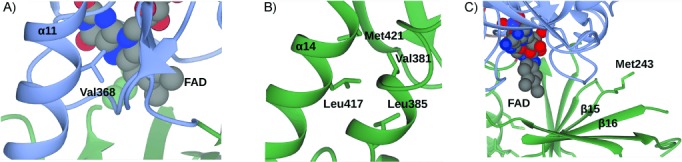
Location of activity hotspots in HbpA as identified by directed-evolution experiments by Schmid, Witholt and co-workers.[24–26] A) Detail of HbpA showing the location of Val368, the mutation of which to Ala resulted in improved activity towards guaiacol. B) Detail showing the location of Leu417, the mutation of which to Phe also resulted in improved activity towards guaiacol. C) Detail of HbpA showing the location of Met243, adjacent in sequence to Ile244, the mutation of which to Val resulted in improved activity towards 2HBP, 2-*sec*-butylphenol and guaiacol. Domains are coloured as in Figure 1.

In the absence of structure, HbpA was subjected to directed-evolution experiments that resulted in mutants capable of hydroxylating 2-*tert*-butylphenol,[24,25] guaiacol[25] and indole.[26] The structure of HbpA allows the sites of mutations in these variants to be located for the first time. The variant Val368Ala/Leu417Phe was reported to have eightfold improved catalytic efficiency towards guaiacol. Another variant, Ile244Val displayed higher activity towards 2-*sec*-butylphenol, guaiacol and 2-HBP. A model of HbpA constructed by using the structure of PHHY as a template, placed Val368 and Leu417 near the surface of the HbpA monomer, and within the substrate binding pocket.[23] Val368 in each subunit is indeed at the periphery of the HbpA tetramer in helix α11 within the flavin binding domain **D1** (Figure 4 A), and superimposes with Ile412 of PHHY, but is approximately 13 Å from the FAD, with the loop formed by residues 317–324 between them. Leu417 is one of the first residues in the long helix α14 that helps to form the larger substrate binding domain **D2** and is actually 27 Å distant from the FAD molecule (Figure 4 B) Leu417 superimposes in the region of Met438 of PHHY, rather than Val480, as first thought.[23] Here it makes stabilising hydrophobic interactions with Val381, Leu385 and Met421, which support the β-sheet underlying the substrate-binding pocket.

Electron density for Ile244 is absent in the FAD-complex structure, but the adjacent residue, Met243, places Ile244 within the loop that separates strands β15 and β16 in the β-sheet underlying the substrate binding pocket (Figure 4 C), and superimposing with residue Leu285 in PHHY, not with the catalytic residue Tyr289 of PHHY within the substrate binding pocket, as previously suggested.[23] As each of the three mutated residues is too distant from the FAD binding site to be implicated in catalysis directly, other roles in substrate transport or active-site access must be considered. Val368 is on helix α11, which corresponds to region 354–363 in RebC, which is proposed to act as the gateway for substrate and product in that enzyme. In HbpA, helix α11 forms one side of a hydrophobic channel, the opening of which also includes the mobile loop 195–203, as revealed by the apo structure. The mobility in this region of the protein could mean that it serves as a gateway in HbpA for entry of the aromatic substrate.

## Conclusions

Flavoprotein aromatic hydroxylases are promising candidates for roles in both the preparative hydroxylation of aromatic substrates and in bioremediation processes. The determination of their structure is a significant advance in being able to interpret the results of in vitro evolution experiments and in informing new protein-engineering experiments. In addition to providing the first structural perspective on mechanism and specificity in HbpA, the structure also reveals new avenues for rational engineering experiments that could be targeted towards improving or altering its activity.

## Experimental Section

**HbpA gene cloning, expression and protein purification**: The gene encoding HbpA was provided by Bartlomiej Tomaszewski (Technical University of Dortmund, Germany). The gene was subcloned into the pET-YSBLIC-3C vector, in which cloned genes become equipped with a sequence encoding an N-terminal His_6_ tag. The HbpA gene was amplified by PCR from the template plasmid by using the primers 5′-CCAGG GACCA GCAAT GTCGA ATTCT GCAGA AACTG ATGTT CTTAT TGTGG-3′ (forward) and 5′-GAGGA GAAGG CGCGT TACGC CCTCC CAAGG ATGCT CTTCA C-3′ (reverse). Following agarose gel analysis of the PCR product, the relevant band was eluted from the gel by using a PCR Cleanup kit (Qiagen). The gene was then subcloned into the pET-YSBLIC-3C vector according to published techniques.[35] The recombinant plasmid was used to transform *E. coli* XL1-Blue cells (Novagen), yielding colonies that, in turn, gave plasmids by standard miniprep procedures; these were sequenced to confirm the identity and sequence of the gene.

The recombinant plasmid containing the HbpA was used to transform *E. coli* BL21(DE3) cells by using kanamycin (30 μg mL^−1^) as antibiotic marker on lysogeny broth (LB) agar. Single colonies from an agar plate grown overnight were used to inoculate cultures of LB (5 mL), which were then grown overnight at 37 °C with shaking at 180 rpm. The starter cultures served as inocula for 1 L cultures of LB in which cells were grown until the optical density (OD_600_) had reached a value of 0.8. Expression of HbpA was then induced by the addition of isopropyl β-D-1-thiogalactopyranoside (IPTG, 1 mm). The cultures were then incubated at 18 °C in an orbital shaker at 180 rpm for approximately 18 h. The cells were harvested by centrifugation for 15 min at 4225 *g* in a Sorvall GS3 rotor in a Sorvall RC5B Plus centrifuge.

Cell pellets were resuspended in Tris**⋅**HCl buffer (20 mL, 50 mm, pH 7.5) containing NaCl (300 mm; henceforth referred to as “buffer A”), per litre of cell culture. The cell suspensions were then sonicated for 10*×*45 s bursts at 4 °C with 30 s intervals. The soluble and insoluble fractions were separated by centrifugation for 30 min at 26 892 *g* in a Sorvall SS34 rotor. The crude cell lysate from 1 L cell culture was filtered and then loaded onto a 5 mL HisTrap FF crude column (GE Healthcare), which was washed with buffer A. The column was eluted with imidazole (linear gradient of 20–300 mm) over 20 column volumes at a flow rate of 2.5 mL min^−1^. Fractions containing HbpA, as determined by SDS-PAGE analysis were combined, and the volume was reduced to yield a protein concentration of 10 mg mL^−1^. The concentrated protein was a deep yellow colour, suggestive of bound oxidised FAD within the protein.

**Protein crystallisation**: Crystallisation conditions for HbpA were determined by using a Mosquito robot (TTP Labtech, Melbourn, UK) in conjunction with commercially available screens. Trials were conducted in 96-well plates with sitting-drop format by using 300 nL drops (150 nL protein plus 150 nL precipitant solution). Positive hits were scaled up in 24-well Linbro dishes by using the hanging-drop method of crystallisation, with crystallisation drops containing protein solution (1 μL) and precipitant reservoir (1 μL). The best HbpA crystals were obtained in drops that contained PEG 3350 (18 %, *w*/*v*), KSCN (0.15 m) in Bis**⋅**Tris propane buffer (pH 5.5), with a protein concentration of 10 mg mL^−1^. Despite the yellow colour of the protein solution and of the crystals, the occupancy of flavin was found to be poor in the active sites of the solved structure; this led to the apo structure described *above*. In order to obtain a structure with bound FAD, it therefore proved necessary to soak the HbpA crystals in a solution of the mother liquor with added FAD (1 mm), as well as 20 % ethylene glycol (as cryoprotectant) for 5 min prior to flash-cooling in liquid nitrogen for X-ray diffraction analysis. Crystals were tested for diffraction by using a Rigaku Micromax-007HF X-ray generator fitted with Osmic multilayer optics and a mar345 imaging plate detector (Marresearch, Norderstedt, Germany). Those crystals that diffracted to greater than 3 Å resolution were retained for full dataset collection at the synchrotron.

**Data collection, structure solution, model building and refinement of HbpA**: Complete datasets for apo-HbpA and its FAD complex were collected on beamlines I04 and I04-1, respectively, at the Diamond Light Source (Didcot, UK). Data were processed and integrated by using XDS[36] and scaled by using SCALA[37] within the Xia2 processing system.[38] Data collection statistics are given in Table 2. The structure of HbpA was solved by using BALBES,[39] which selected a monomer of structure PDB ID 3IHG, the aklavinone-11-hydroxylase,[17] as a search model. The solution contained four molecules in the asymmetric unit; this represents one tetramer. The structures were built and refined by using iterative cycles of Coot[40] and REFMAC[41] employing local NCS restraints in each case. For the FAD complex of HbpA, the omit maps, after building and refinement of the protein backbone and side chains, revealed clear residual density at the active sites, which was successfully modelled as FAD. The final structures of apo*-*HbpA and the FAD complex had *R*_cryst_/*R*_free_ values of 19.1/24.1 and 17.0/20.3, respectively. The structures were validated by using PROCHECK.[42] Refinement statistics are presented in Table 2. The Ramachandran plot for apo-HbpA showed 93.5 % of residues to be situated in the most favoured regions, in addition 5.6 % were allowed and 0.9 % were outlier residues. For the FAD complex, the respective values were 95.9, 3.4 and 0.7 %. Coordinates and structure factors for both apo-HbpA and the FAD complex have been deposited in the Protein Data Bank with the accession codes 4CY6 and 4CY8, respectively.

**Table 2:** Data collection and resolution statistics for apo-HbpA and the HbpA–FAD complex.

	apo*-*HbpA	HbpA-FAD complex
beamline	Diamond I04	Diamond I04-1
*λ* [Å]	0.97949	0.91999
resolution [Å]	85.97–2.76 (2.83–2.76)	59.53–2.03 (2.08–2.03)
space group	*P*1	*P*1
unit cell		
*a*, *b*, *c* [Å]	79.35, 94.67, 99.16	80.00, 96.18, 102.16
*α*, *β*, *γ* [°]	115.03, 96.09, 109.59	114.09, 95.80, 109.31
no. of molecules in the	4	4
asymmetric unit		
unique reflections	59 321 (4372)	146 673 (11 110)
completeness [%]	97.9 (97.8)	90.0 (92.0)
*R*_merge_ [%]	0.10 (0.48)	0.07 (0.43)
*R*_p.i.m._	0.10 (0.36)	0.05 (0.35)
multiplicity	2.1 (2.0)	2.3 (2.4)
〈*I*/*σ*(*I*)〉	5.4 (1.6)	7.4 (2.1)
CC_1/2_	0.99 (0.70)	1.00 (0.69)
overall *B* factor from the	63	38
Wilson plot [Å^2^]		
*R*_cryst_/*R*_free_ [%]	19.1/24.1	17.0/20.3
rmsd 1–2 bonds [Å]	0.010	0.016
rmsd 1–3 bonds [°]	1.42	1.76
average main chain *B* [Å^2^]	60	35
average side chain *B* [Å^2^]	64	40
average water *B* [Å^2^]	48	40

**Site-directed mutagenesis**: The mutants of HbpA, His48Ala, Asp117Ala and Arg242Ala were generated by using a Clontech InFusion kit, according to the manufacturer's instructions, from the primers listed in Table 3. Mutants genes were expressed, and proteins were purified according to the methods described above for the wild-type.

**Table 3:** Primers used to generate site-directed mutants of HbpA.

Variant	Primer	
His48Ala: For	5′-AGGTC GGCGA TTATC AATCA GCGCA CGATG GAAAT	
	TCTG-3′	
His48Ala: Rev	5′-AGGTC GGCGA TTATC AATCA GCGCA CGATG GAAAT	
	TCTG-3′	
Asp117Ala: For	5′-TACTG TGCGT TGCCG CAGTT GTATT TTGAG CCGAT	
	GGTG-3′	
Asp117Ala: Rev	5′-CGGCA ACGCA CAGTA TCTTG ATGGG CTTGC CAGCT	
	C-3′	
Arg242Ala: For	5′-GCGCT GGCGA TGATT CGGCC CTGGA ATAAG TGGAT	
	TTG-3′	
Arg242Ala: Rev	5′-AATCA TCGCC AGCGC CGCGA CGCCG ACACC	ATTG-3′

**Enzyme assay**: Rates of NADH oxidation both without and with 2HBP (the latter for the purposes of determining *K*_m app_ values for HbpA variants) were determined by UV spectrophotometry using the method of Meyer et al.[25] HbpA variants were also assayed for their ability to oxygenate 2HBP, and the reactions were analysed by HPLC. Each assay contained 2HBP (0.5 mm), NADH (1.5 mm), air-saturated phosphate buffer (20 mm, pH 7.5) and purified HbpA variant (1 mg mL^−1^) in a total volume of 2 mL. Product evolution was followed in a time-dependent manner; samples (250 μL) were taken, and the reaction was stopped by the addition of 10 % perchloric acid (8 μL) after 0, 2, 4, 8 and 24 h. The resulting precipitate was removed by centrifugation at 16 300 *g*, for 1 min. The supernatant was then diluted 1:1 with methanol containing phosphoric acid (0.1 %, *w*/*v*). The samples were analysed by HPLC using a Phenomenex Aqua 3 μm C18 125A column at 40 °C and eluting at a flow rate of 200 μL min^−1^ with buffer A (20 % acetonitrile, 80 % sodium acetate (50 mm), pH 5.0) and buffer B (80 % acetonitrile and 20 % sodium acetate (50 mm), pH 5.0). The gradient used was as follows: 10–40 % buffer B (0–5 min); 40–100 % buffer B (5 min); 100 % buffer B (5–15 min); 100–10 % buffer B (15 min); 10 % buffer B (15–25 min). 2HBP eluted at *t*_R_=11.1 min, and the product 2,3-dihydroxybiphenyl eluted at *t*_R_=10.0 min. The substrate and product concentrations were calculated from calibration curves obtained under the same conditions. All samples were run in duplicate.

**Docking**: Automated docking was performed by using AutoDock Vina 1.1.2.[43] A monomer structure of the FAD complex of HbpA was prepared by using AutoDock utility scripts. Coordinates for the product 23DHBP (**4**) were prepared by using PRODRG.[44] The active site of HbpA was contained in a grid of 30×32×24 with 0.375 Å spacing, centred around the catalytic centre (co-ordinates: −9.947, 34.123, −16.228) which was generated using AutoGrid in the AutoDock Tools interface. The dockings were performed by Vina, therefore the posed dockings were below 2 Å rmsd. The results generated by Vina were visualised in AutoDock Tools 1.5.6 where the ligand conformations were assessed both upon lowest Vina energy, but also according to criteria established by previous studies on mechanism and substrate selectivity in this enzyme class (vide supra).
